# Extracorporeal Blood Purification Therapy to Deal a Deferasirox Induced Life‐Threatening Hepatic Encephalopathy in a Septic Child With Sickle‐Cell Disease: A Case Report

**DOI:** 10.1002/jca.70032

**Published:** 2025-05-22

**Authors:** E. Rossetti, A. Cappoli, R. Labbadia, G. Leone, F. Chiusolo, F. Tortora, D. Martinelli, M. Marano

**Affiliations:** ^1^ Pediatric Intensive Care Unit, Anaesthesia and Intensive Care Division Bambino Gesù Children's Hospital, IRCCS Rome Italy; ^2^ Unit of Nephrology and Dialysis, Division of Pediatric Subspecialties Bambino Gesù Children's Hospital, IRCCS Rome Italy; ^3^ Unit of Transfusion Medicine, Division of Diagnostic and Laboratory Medicine Bambino Gesù Children's Hospital, IRCCS Rome Italy; ^4^ Division of Metabolism Bambino Gesù Children's Hospital, IRCCS Rome Italy; ^5^ Area Rossa Division Pediatric Poison Control Center, Bambino Gesù Children's Hospital, IRCCS Rome Italy

**Keywords:** continuous renal replacement therapy, deferasirox, encephalopathy, hyperammonemia, liver failure, plasma exchange, sepsis

## Abstract

This report details a rare pediatric case of hyperammonemic encephalopathy caused by the oral iron chelating drug deferasirox (DFR) in a septic patient. It is our contention that this study lends support to the existing literature, as it describes the case of a 15‐year‐old female patient with a history of sickle‐cell disease who presented with a fever and vomiting, rapid development of sleepiness, consciousness disturbances, medium mydriasis, neck stiffness, and trismus with seizure. Her Glasgow Coma Scale (GCS) score was 5. Laboratory tests revealed an increase in creatinine, metabolic acidosis, hyperammonemia, high cerebrospinal fluid (CSF) glutamine levels, alterations in coagulation and in liver function, rising inflammatory markers, cerebral oedema on brain Computerized Tomography (CT) scan, 10^6 copies/ml of Methicillin‐Resistant *Staphylococcus Aureus* (MRSA) in pulmonary swab film array, and elevated DFR blood level. The treatment plan involved the early cessation of DFR, the correction of acidosis, mechanical ventilation, mannitol and bioarginine, vasoactive drug, antibiotics, and supportive care with continuous veno‐venous hemodiafiltration (CVVHDF) for hyperammonemia and therapeutic plasma exchange (TPE) for a high CSF glutamine level resulting from cytotoxic encephalopathy. The patient successfully overcame the multiorgan failure, with no permanent neurologic complications. It is our opinion that healthcare providers and family caregivers of patients with chronic disease may be particularly attuned to the emergence of any sign or symptom, and thus well positioned to take prompt action to avert life‐threatening clinical deterioration due to rising DFR levels. It is recommended that critical care providers commence extracorporeal blood purification therapies (EBPT) at the earliest opportunity, taking care to adapt the technique to the specific needs of the patient and to avoid the potential for fatal neurological complications.

## 1 Case Report

This report details a rare pediatric case of hyperammonemic encephalopathy caused by the oral iron chelating drug deferasirox (DFR) in a septic patient. This 15‐year‐old female patient had a past history of sickle‐cell disease and had undergone multiple blood transfusions on an annual basis since early childhood. She was taking a daily dose of 1080 mg of oral DFR (20 mg/kg) when she developed a fever, nausea, and vomiting during a sailing stage on a summer holiday in 2023. Consequently, she immediately discontinued the DFR treatment and commenced treatment with anti‐emetic drugs at home. Nevertheless, her condition deteriorated abruptly overnight, presenting with severe encephalopathy, seizures, neck rigidity, and mydriasis, necessitating transfer to our PICU.

Her Glasgow Coma Scale (GCS) score was 5 and DFR blood level was 205 μg/mL (normal value of < 50 μg/mL) at the time of admission to the PICU.

Laboratory tests revealed an increase in creatinine, metabolic acidosis, hyperammonemia, alterations in coagulation and liver function, rising inflammatory markers, and cerebral oedema on brain Computerized Tomography (CT) scan: furthermore, she required vasoactive drug support with noradrenaline infusion at a rate of 0.3 mcg/kg/min.

An early examination of the CSF yielded negative results for bacteria and viruses. Blood cultures, virus molecular, and serological tests were negative too. The source of sepsis was identified as pneumonia due to MRSA in the pulmonary swab film array, with a concentration of 10^6^ copies/ml. Furthermore, metabolic and genetic evaluations ruled out urea cycle disorders in this patient.

**FIGURE 1:**
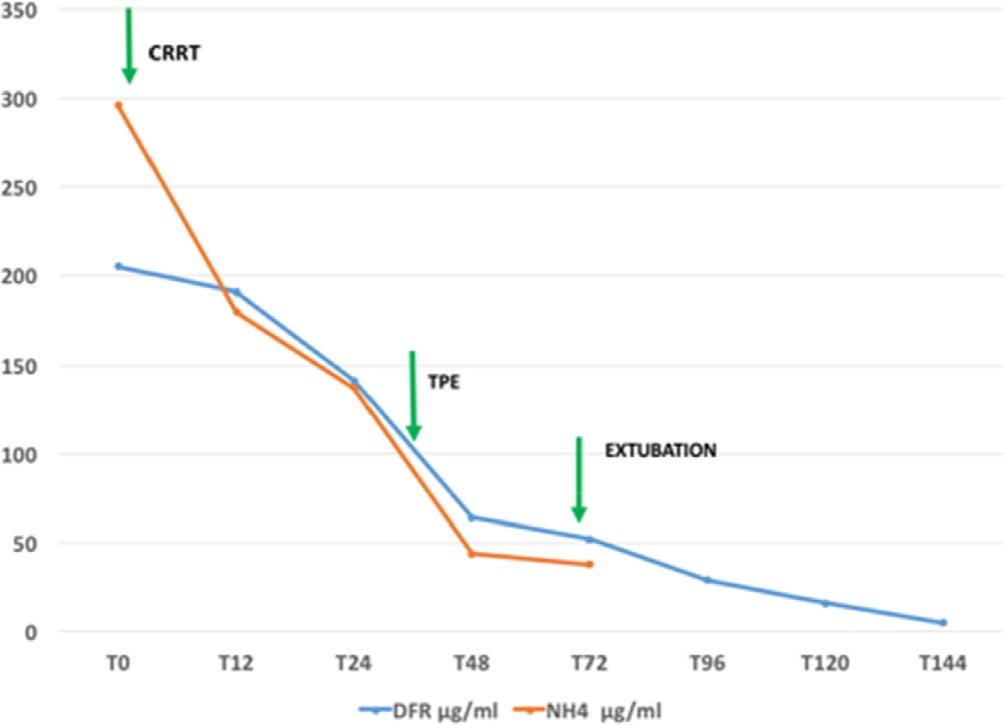
DFR and NH4 trend.

At the outset, the patient was supported with mechanical ventilation for pneumonia, scavenger ammonium drugs treatment with bioarginine and sodium benzoate, and continuous renal replacement therapy (CRRT) to reduce hyperammonemia and acidosis, and mannitol to address cerebral oedema. Indeed, within the initial 36‐h period, she demonstrated a recovery from acidosis, renal, and liver failure, while the administration of vasoactive drugs was terminated within the first 24 h of intensive care. Despite prompt ammonium reduction (Figure 1) with CRRT during the initial 24 h of PICU stay, her neurological state did not improve. On the third day of PICU stay, a markedly elevated CSF glutamine concentration of 3340 μM/L (normal range 284–566) was identified through amino acid analysis, and the brain MRI scan corroborated the presence of cytotoxic oedema. Furthermore, the electroencephalogram (EEG) demonstrated a slow pattern, and the Babinski reflex was positive bilaterally.

Thus, in order to avoid fatal brain damage due to DFR administration as described in other cases in current literature according to Towermann AS [1] and Braga CCB et al. [2], we followed critical care providers experience in the treatment of pediatric ALF and severe HE over the past decade: the use of hybrid EBPT through CRRT and TPE as an effective technique in improving acute HE due to liver failure of unknown etiology [3, 4].

The two devices were connected in parallel by placing the TPE withdrawal and return lines on the withdrawal lumen of the CRRT. The only parameter modified was the withdrawal speed of the CRRT: it was set to 20 mL/min higher than the withdrawal speed of the TPE.

We used COMTEC Fresenius BCT and performed plasma exchange for three consecutive days: it required replacing 1.5 volumes of plasma (4400 mL) with albumin 5% (70%, 3300 mL) and fresh frozen plasma (30%, 1200 mL). Based on our procedure, hypocalcemia was corrected according to the blood gas analyzer values for ionized calcium lower than 1 mmol/L.

Hence, the patient demonstrated a rapid improvement in both her EEG and neurological status following the initial course of TPE. She was weaned off mechanical ventilation and CRRT, and her condition improved further with each subsequent course of TPE in addition to CRRT, until she was fully awake. The patient was transferred to the pediatric ward on the sixth day of the PICU stay, exhibiting normal renal, liver, and neurological function, and has been discharged from the hospital 8 days later.

## 2 Discussion

The long‐term administration of DFR in children with transfusion‐dependent thalassemia and sickle cell disease [5] may result in the development of hyperammonemia and liver failure with HE. This is thought to be due to the depletion of intramitochondrial bicarbonate or the disruption of other pivotal urea cycle molecules.

In their report, Ribas et al. [6] describe the occurrence of hyperammonemia in primary and secondary mitochondrial disorders due to inhibition of mitochondrial oxidative phosphorylation.

Martinelli D et al. [7] have described this uncommon complication of DFR treatment. Furthermore, several factors may affect DFR bioavailability through differences in drug absorption and first‐pass effect, such as bowel transit time, lipid solubility, or anionic organic carriers due to pharmacogenomics polymorphisms and half‐life drug variations [8].

Moreover, the package insert for DFR was updated, recommending a reduction in dosage for ferritin levels below 1000 ng/mL, cessation of treatment for levels below 500 ng/mL, and the interruption of treatment when patients are volume depleted until volume status and renal function normalize [9].

As posited by Ilhuicamina et al., hyperammonemia and hepatic failure induce alterations in glutamatergic neurotransmission, which may be the primary cause of HE [10].

A limitation of this report is the lack of data regarding cerebrospinal fluid (CSF) level following TPE and the patient's subsequent awakening.

Any stressful event may induce hemolysis among patients with sickle cell disease. On DFR treatment, the stressful event can be anyone (sepsis, dehydration, excessive tiredness, etc.): a sudden hyperammonemic encephalopathy may appear and be mistaken for sepsis.

It is our contention that this patient underwent dehydration during the sailing stage of the summer period, which was accompanied by a relative hypovolemic state and an exacerbation of the effects of DFR chelation in the context of iron deprivation: after therapeutic oral dose DFR's typical half‐life is 8–16 h. Hence, we suppose the ferritin blood level should be very low at first. On the following hours, the patient exhibited hyperammonemic symptoms, manifesting as nausea and vomiting, which prompted the immediate cessation of DFR administration. The patient's septic state resulted in a rise in ferritin level later, while the DFR blood level was higher than our initial evaluation upon admission to the PICU. Thus, she developed liver and renal failure with acidosis and hyperammonemia, which enhanced glutamine production and significantly impaired brain function. Excessive hyperammonemia and glutamine have been identified as triggers of neuronal damage. Furthermore, septic shock introduced an additional clinical challenge due to hemodynamic instability.

In this sickle cell patient, liver function exams and ammonium blood level are supposed to be scheduled regularly, and a planned DFR serum level is recommended (normal value 0.16–40 mcg/ml).

As critical care providers, we should think to start earlier CRRT and TPE, even while the identification of the underlying disease process is ongoing, and tailor EBPT to the patient in order to optimize effective brain‐saving management.

## Consent

Was obtained from the minor's parents in accordance with national legislation and institutional requirements.

## Conflicts of Interest

The authors declare no conflicts of interest.

## Data Availability

The data that support the findings of this study are available on request from the corresponding author. The data are not publicly available due to privacy or ethical restrictions.

## References

[1] A. S. Towerman , K. P. Guilliams , R. Guerriero , et al., “Hyperammonemia and Acute Liver Failure Associated With Deferasirox in Two Adolescents With Sickle Cell Disease,” British Journal of Haematology 201, no. 4 (2023): e30–e33, 10.1111/bjh.18770. 36964994

[2] C. C. B. Braga , B. D. Benites , D. M. de Albuquerque , et al., “Deferasirox Associated With Liver Failure and Death in a Sickle Cell Anemia Patient Homozygous for the ‐1774delG Polymorphism in the Abcc2 Gene,” Clinical Case Reports 5, no. 8 (2017): 1218–1221, 10.1002/ccr3.1040. 28781827 PMC5538070

[3] E. Rossetti , F. Polisca , F. Tortora , R. Bianchi , and S. Picardo , “Early Hybrid Extracorporeal Therapies in Pediatric Acute Liver Failure of Unknown Etiology,” Blood Purification 49, no. 3 (2020): 382–384, 10.1159/000504559. 31910419

[4] E. C. Alexander and A. Deep , “Therapeutic Plasma Exchange in Children With Acute Liver Failure (ALF): Is It Time for Incorporation Into the ALF Armamentarium?,” Pediatric Nephrology (Berlin, Germany) 37, no. 8 (2022): 1775–1788, 10.1007/s00467-021-05289-0. 34647173 PMC9239959

[5] A. Ramaswami , D. J. Rosen , J. Chu , B. Wistinghausen , and R. Arnon , “Fulminant Liver Failure in a Child With β‐Thalassemia on Deferasirox: A Case Report,” Journal of Pediatric Hematology/Oncology 39, no. 3 (2017): 235–237, 10.1097/MPH.0000000000000654. 27479018

[6] G. S. Ribas , F. F. Lopes , M. Deon , and C. R. Vargas , “Hyperammonemia in Inherited Metabolic Diseases,” Cellular and Molecular Neurobiology 42, no. 8 (2022): 2593–2610, 10.1007/s10571-021-01156-6. 34665389 PMC11421644

[7] D. Martinelli , B. M. Goffredo , F. S. Falvella , and M. Marano , “Acute Hyperammonemia in Children Under Deferasirox Treatment: Cutting the Gordian Knot,” Clinical Toxicology 57, no. 5 (2019): 375–377, 10.1080/15563650.2018.152342. 30442064

[8] D. Chirnomas , A. L. Smith , J. Braunstein , et al., “Deferasirox Pharmacokinetics in Patients With Adequate Versus Inadequate Response,” Blood 114, no. 19 (2009): 4009–4013, 10.1182/blood-2009-05-222729 Epub 2009 Sep 1. 19724055 PMC2774541

[9] Jadenu(deferasirox) (Novartis AG, 2020).

[10] D. L. Ilhuicamina , I. D. Limón , I. Angulo‐Cruz , L. Sánchez‐Abdon , and A. Patricio‐Martínez , “Disturbance of the Glutamate‐Glutamine Cycle, Secondary to Hepatic Damage, Compromises Memory Function,” Frontiers in Neuroscience 15 (2021): 578922, 10.3389/fnins.2021.578922. 33584185 PMC7873464

